# Neutral markers reveal complex population structure across the range of a widespread songbird

**DOI:** 10.1002/ece3.11638

**Published:** 2024-07-07

**Authors:** Aaron Veale, Matthew W. Reudink, Theresa M. Burg

**Affiliations:** ^1^ Department of Biological Sciences University of Lethbridge Lethbridge Alberta Canada; ^2^ Department of Biological Sciences Thompson Rivers University Kamloops British Columbia Canada

**Keywords:** bluebird, gene flow, population genetics, RADseq, Sialia, SNP

## Abstract

Understanding how both contemporary and historical physical barriers influence gene flow is key to reconstructing evolutionary histories and can allow us to predict species' resilience to changing environmental conditions. During the last glacial maximum (LGM), many high latitude North American bird species were forced into glacial refugia, including mountain bluebirds (*Silia currucoides*). Within their current breeding range, mountain bluebirds still experience a wide variety of environmental conditions and barriers that may disrupt gene flow and isolate populations. Using single nucleotide polymorphisms (SNPs) obtained through restriction site‐associated DNA sequencing, we detected at least four genetically distinct mountain bluebird populations. Based on this structure, we determined that isolation‐by‐distance, the northern Rocky Mountains, and discontinuous habitat are responsible for the low connectivity and the overall history of each population going back to the last glacial maximum. Finally, we identified five candidate genes under balancing selection and three loci under diversifying selection. This study provides the first look at connectivity and gene flow across the range of these high‐altitude and high latitude songbirds.

## Graphical abstract

Understanding how both contemporary and historical physical barriers influence gene flow is key to reconstructing evolutionary histories and can allow us to predict species' resilience to changing environmental conditions. Using single nucleotide polymorphisms obtained through restriction site‐associated DNA sequencing, we detected at least four genetically distinct mountain bluebird populations. Based on this structure, we determined that isolation‐by‐distance, the northern Rocky Mountains, and discontinuous habitat are responsible for the low connectivity, and the overall history of each population going back to the last glacial maximum.

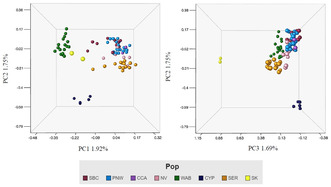

## 1 INTRODUCTION

Population genetics allows for the distinction of breeding populations, the determination of potentially unique genetic units, and the assessment of their level of connectivity. Compared with large, interconnected populations, small, isolated breeding groups may be at increased risk of extirpation due to environmental or demographic stochastic events (Fordham et al., 2022; Frankham, 2005; Lande, 1993; Melbourne & Hastings, 2008), and some populations can be more sensitive to changes than others (Fraser et al., 2004; Wittmer et al., 2007). One method of understanding how a species copes with large changes in population size is to analyze gene flow along with the historical biogeography of its range (Dayton & Szczys, 2021; Ony et al., 2021). For example, a species with a historically large, continuous habitat may see sharp population decreases and changes to behaviors, when their habitat is suddenly fragmented (Peery et al., 2010). Therefore, to better understand breeding population connectivity, we can use genetic variation both within and among populations to measure the temporal and spatial changes in allele frequencies (Moore, 1977).

Two key factors influencing population structure are a species capacity for dispersal and the extent of population connectivity (Slatkin, 1985). Reduced gene flow in vagile species can occur when their range is divided by geographic features such as mountain ranges, fragmented habitat, or water bodies (see Sánchez‐Montes et al., 2018; Vignieri, 2005). Selective pressures experienced by different populations may also vary, leading to local adaptation (e.g., Bradshaw & Holzapfel, 2001; Savolainen et al., 2007). Geographic features, such as mountain ranges, have sustained effects on both paleohistorical and contemporary gene flow. One of the most influential barriers to gene flow in many species was the Pleistocene glaciations and the creation of disjunct habitat leading to isolated populations, which has left a genetic footprint in a number of high latitudes species (Arcones et al., 2021; Bagley et al., 2020; Graham & Burg, 2012; van Els et al., 2012).

Starting ~2.58 million years before present (myBP), global temperatures decreased, and ice sheets expanded at higher latitudes in both the northern and southern hemispheres (Gibbard & Head, 2009). During the Pleistocene (2.58 myBP–11.7 kyBP), the planet experienced repeated cycles of cooler periods with glacial expansion, and warmer interglacial periods. North America was dominated by three expansive ice sheets ~25 kyBP (Dalton et al., 2023): the Laurentide ice sheet east of the Rocky Mountains to the Atlantic Coast of Canada, the Innuitian ice sheet in the far north and the Cordilleran ice sheet west of the Rocky Mountains to the Pacific Coast (Blaise et al., 1990; Dalton et al., 2022; Dyke, 2004; Dyke et al., 2002). Many species were forced to retreat into isolated pockets of suitable habitat known as refugia (Haffer, 1969), with genetic data supporting the presence of multiple northern refugia including Beringia and the Pacific Northwest for plants (Abbott et al., 2000; Abbott & Comes, 2004; Anderson et al., 2006; Brunsfeld et al., 2001; Shafer et al., 2010; Soltis et al., 1997; Thompson & Whitton, 2006; Tremblay & Schoen, 1999), birds (Barrowclough et al., 2004; Lait & Burg, 2013; van Els et al., 2012), and mammals (Brunsfeld et al., 2001; Leonard et al., 2000; Steppan et al., 1999). As the ice sheets retreated, taxa in these refugia colonized newly opened habitat leading to secondary contact between previously isolated lineages (Latch et al., 2009; Omland et al., 2000).

**FIGURE 1:**
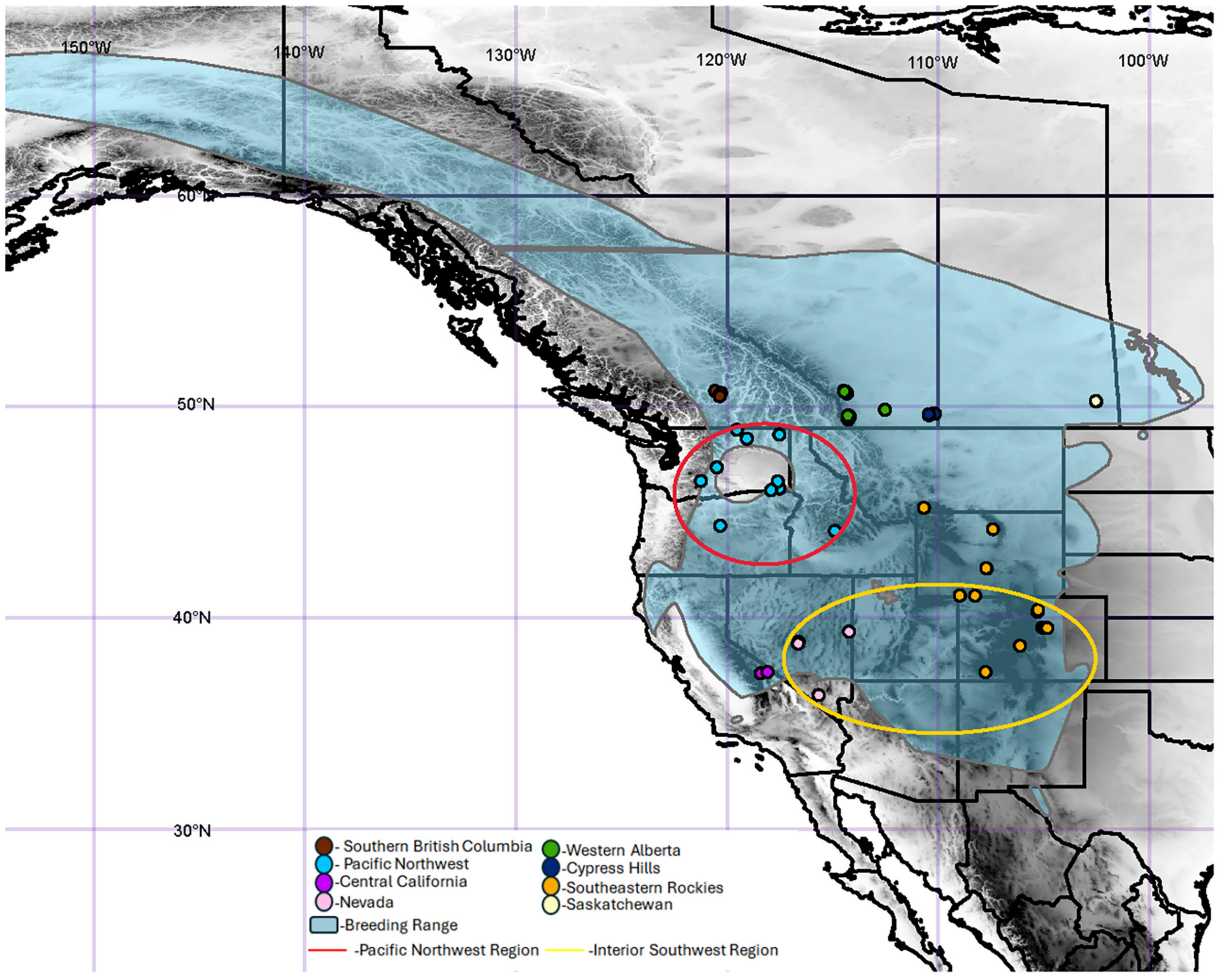
Map of North America overlayed with the breeding range of the mountain bluebird. Areas where breeding occurs but is scarce (as described by Johnson & Dawson, 2020) are separated by the purple line in northern British Columbia. Although our southern Nevada samples fall outside of the range map, they were collected during the breeding season. Possible glacial refugia circled in the Pacific Northwest (red) and the Interior Southwest (yellow). Range map provided by Birdlife International (2021).

While the population structure has been assessed and areas of secondary contact have been proposed for many high‐latitude (e.g., Lait & Burg, 2013; Milot et al., 2000; van Els et al., 2012) and high‐elevation (e.g., Hindley et al., 2018; Spellman et al., 2007) breeding songbirds, few studies have analyzed migratory species that breed at both high latitudes and high elevations simultaneously. Likewise, where exactly suitable habitat existed during the LGM still remains unknown for many species, including the mountain bluebird (*Sialia currucoides*). The mountain bluebird is a partially migratory songbird whose current range includes large portions of previously glaciated areas in western North America as far north as central Alaska (but is listed as being scarce in northern British Columbia upwards), east to central Manitoba, and south to New Mexico (Johnson & Dawson, 2020) (Figure 1). Currently, there is no known phenotypic variation within mountain bluebirds and no described subspecies (Johnson & Dawson, 2020). Mountain bluebirds are secondary cavity nesters and can adapt to a range of habits (Medin et al., 2000). However, they achieve their highest densities in mixed conifer‐alpine environments (Medin et al., 2000) and stands of trembling aspen (*Populus tremuloides*) (Johnson & Dawson, 2020). For example, in the Cypress Hills of Alberta and Saskatchewan, relic populations of lodgepole pine (*Pinus contorta*), trembling aspen (*Populus tremuloides*), and white spruce (*Picea glauca*) provide ample nesting sites (Sauchyn, 1990). Mountain bluebirds are associated with high‐elevation environments, typically nesting between 1500 and 3800 m a.s.l. (Johnson et al., 2007, 2013), but have been known to consistently nest as low as ~800 m (Morrison et al., 2014) and as high as ~4270 m (Herlugson, 1981). Through the widespread use of nest boxes, mountain bluebirds have increased in abundance in grasslands and savannah‐like habitats where fewer trees would have severely limited nesting sites (Johnson & Dawson, 2020).

Mountain bluebirds offer a unique combination of life history traits: They breed at both high elevation and high latitude, most populations migrate, and they arrive early on the breeding grounds when conditions may still be harsh (Power & Lombardo, 1996). Like many high‐latitude and high‐elevation migrants (Couet et al., 2022; Freeman et al., 2018), mountain bluebirds are not immune to the effects of climate change. For example, the arrival dates for northern breeding populations (e.g., those in British Columbia and Alberta) have advanced significantly in the 20th and 21st centuries (Lane & Pearman, 2003; Ranson, unpublished). Range shifts are another phenomenon documented in high‐latitude/high‐elevation breeders (Couet et al., 2022; Freeman et al., 2018; Prytula et al., 2023). While mountain bluebirds themselves have yet to see significant changes to their breeding range, their congenerics (the western bluebird, *Sialia mexicana*, and the eastern bluebird, *Sialia sialis*) have (Duckworth & Badyaev, 2007; Sonnleitner et al., 2022). In some cases, renewed secondary contact may even result in hybridization (e.g., Duckworth & Semenov, 2017) which also impacts genetic diversity (Grant & Grant, 2019).

By using mountain bluebirds as a proxy for similar montane species, we hope to better understand what constitutes a barrier to gene flow. Thus, the primary aim of this study was to determine levels of population structure within mountain bluebird breeding populations. Most adults return to the same breeding site with relatively little movement between breeding seasons (Dawson et al., unpublished in Johnson & Dawson, 2020; Herlugson, 1981; M.W.R., unpublished data). While young birds rarely return to their natal site, they typically disperse a short distance (≤3 km; Dawson et al., unpublished in Johnson & Dawson, 2020). It should be noted, however, that mountain bluebirds do have the propensity for relatively long‐distance travel when renesting; a female (and presumably her mate) traveled over 209 km to renest after a particularly bad storm hit the region (Scott & Lane, 1974). However, this example is notable for how unusual it is in terms of overall distance moved during the breeding season.

Potential philopatry aside, mountain bluebirds also inhabit a fairly extensive breeding range (Figure 1). They appear to be excellent candidates for the isolation‐by‐distance model which posits that increasing geographic distance decreases relatedness of individuals (Wright, 1943). Thus, we predicted reductions in gene flow across the range. Our second aim was to determine in which glacial refugia suitable habitat existed during the LGM and use this as a basis for determining how contemporary and historical geographic barriers influence gene flow. In addition to the Pleistocene ice sheets, other potential barriers continue to persist today (e.g., the Rocky Mountains, discontinuous habitat) and may disrupt the movement of individuals. We used restriction‐site associated DNA sequencing (RADseq) data to determine whether barriers and physical distance restrict gene flow in populations of mountain bluebirds in western North America. In addition, we identified genes under selection in different environments resulting in local adaptation.

## 2 METHODS

### 2.1 Sample collection and DNA extractions

Fieldwork occurred from mid‐May through late June of 2022 to coincide with the mountain bluebird breeding season (Johnson & Dawson, 2020). Birds were located at nest boxes and the location of sampled birds was recorded using a GPS. To sample the birds, we were permitted to use song playback to entice individuals out of their next boxes while mist nests were set on either side. We collected ~50 μL of blood sample (or a rectrix when blood was not available) and took the morphological measurements (e.g., body mass, tarsus length, bill length, wing chord, and weight) of each bird. Samples were stored at ambient temperature in 99% ethanol and then stored at −80°C upon return to the lab. In total, 82 mountain bluebirds were sampled from four locations in Alberta and one site in Saskatchewan. Another 35 samples were acquired from nest boxes around southern Alberta provided by the Mountain Bluebird Trail Society (during nestbox clean out at the end of the breeding season) and 20 from a parentage study provided by Ken Otter (UNBC) and MWR (with 1 chick per nestbox being sampled) from two sites in British Columbia. Finally, muscle tissue samples were obtained from the Denver Museum of Nature and Science (*n* = 15) and the University of Washington Burke Museum (*n* = 96) for a total of 248 samples.

Of the 248 samples acquired, 157 representing 14 geographic sites across western North America were selected based on DNA yield and geographic representation (Figure 1). DNA was extracted using a modified salting‐out DNA extraction (Miller et al., 1988) and normalized to 20 ng/μL prior to library preparation.

### 2.2 RADseq

Libraries were prepared using Peterson et al. (2013) with modifications to include a third enzyme (de Ronne et al., 2023). The inclusion of a third restriction enzyme allows for the cutting of adapter dimers which improves adapter ligation and thus improves the yield of amplified reads (Bayona‐Vásquez et al., 2019). The extracted DNA samples were digested using the restriction enzymes *MspI* (4 base‐pair cutter), *NsiI* (6 base‐pair cutter), and *PstI* (6 base‐pair cutter) following Abed et al. (2019). Library preparation occurred at Université Laval and samples were sequenced on an Illumina NovaSeq 6000 with paired‐end reads at Génome Québec.

### 2.3 Data processing and filtering

Following sequencing, the raw reads were analyzed with Fastqc/0.11.5, generating quality reports for each forward and reverse read to ensure successful sequencing and remove low‐quality sequences with a phred score <30 (Andrews, 2010). We obtained the raw reads from Génome Québec which we demultiplexed with Saber/1.00, and then removed adapter and barcode sequences using Cutadapt (Martin, 2011), rendering the sequences to ~80 bp in length.

An annotated reference genome for the Swainson's thrush (*Catharus ustulatus*) (Termignoni‐Garcia et al., 2022), assembled at chromosome level, was used in the reference‐based pipeline of Stacks. The reference genome was first indexed using samtools/0.1.2 (Danecek et al., 2021). Next, forward and reverse reads were aligned to the reference genome using the Burrows–Wheeler Alignment (bwa) tool 0.7.15 and low‐quality alignments were marked (Li & Durbin, 2009). Samtools/0.1.2 was used to sort and index the resulting binary alignment map (bam) files. Variants were then called using default parameters (–model marukilow, –var‐alpha 0.01, –gt‐alpha 0.05) in the Stacks/2.3e ref_map pipeline. Reads were analyzed with Stacks/2.3e using the default stack depth of 3 to genotype and call variant sites for each individual (Catchen et al., 2013). Population was then used to select nucleotide sites with minimum minor allele frequencies (min‐maf) of 0.01 and maximum observed heterozygosities (max‐obs‐het) of 0.5. Furthermore, only loci present in a minimum of seven populations (p), and at least 80% of the individuals within those populations (*r* .8), were selected for. The resulting vcf was filtered using VCFtools/0.1.16 (Danecek et al., 2011) to remove SNPs with more than 10% missing data and individuals with more than 30% missing data prior to downstream analyses. The filtered dataset contained 53,501 linked SNPs and 80 individuals from eight populations (Figure 1).

The populations function in Stacks was used a second time with additional parameters, namely the *write‐single‐snp* command and a min‐maf of 0.05. The *write‐single‐snp* command selects the first SNP when multiple SNPs are present at the same locus, while the min‐maf is applied to the metapopulation and selects sites that meet or exceed the minimum minor allele threshold. Selecting a single SNP per locus reduces the effects of linkage. Employing the min‐maf parameter eliminates any one‐off alleles that may arise due to sequencing error. Following the second round of filtering, 19,259 SNPs remained in the 80 individuals. As neutral markers are hypothesized to be affected by demographic and evolutionary history of a species (Luikart et al., 2003), an additional data set was produced containing loci that were not under either directional or balancing selection. To find putatively neutral loci, Bayescan v2.1 (Foll & Gaggiotti, 2008) was used to verify the 19,259 SNPs. We ran 100,000 iterations (−n 100,000) with a thinning interval of 10 (−thin 10) and 20 pilot runs (−nbp 20) at a length of 5000 (−pilot 5000). A burn‐in value of 50,000 (−burn 50,000) and prior odds for the neutral model (−pr_odds) set to 700. We determined 6998 loci to be neutral based off a log *q*‐value set to >0.0001. The other 12,258 markers were under balancing selection and three were under diversifying selection.

### 2.4 Population analyses with neutral markers

Mean observed heterozygosity and mean expected heterozygosity along with pairwise *F*_ST_ values and the corresponding *p*‐values were calculated in Arlequin v3.5.2.2 (Excoffier & Lisher, 2010). A Benjamini–Hochberg correction was applied to the *p*‐values to account for multiple comparisons (Benjamini & Hochberg, 1995).

We constructed an ancestry matrix using STRUCTURE 2.3.4 (Pritchard et al., 2000). We used a burnin value of 100,000 and a Markov‐chain Monte Carlo (MCMC) value of 300,000 and ran STRUCTURE for *K* = 1–6, 10 iterations for each value of *K*. The subsequent results were collected and ran with STRUCTURE HARVESTER v.0.6.94 (Earl & vonHoldt, 2012). Finally, the plots were visualized using a Clumpak server (Kopelman et al., 2015).

Lastly, a principal coordinate analysis (PCoA) was performed to visualize genetic distances and population structure within the dataset. A genetic distance matrix was created using the pairwise Euclidean distances between each individual in Adegenet package in R‐Studio/2021.09.0 (Jombart & Ahmed, 2011). We used GenAlEx v6.5 (Peakall & Smouse, 2006, 2012) to visualize and create the PCoA using the Euclidean distance matrix.

### 2.5 Isolation‐by‐distance with neutral markers

To test for isolation‐by‐distance, a Mantel test was performed with 99 permutations using GenAlEx v6.5 to determine whether there was a significant correlation (*p* < .05) between geographic distance and genetic distance. We used the same Euclidean distances calculated for the PCoA. The pairwise geographic distance matrix between all 80 individuals was generated in GenAlEx v6.5 using sample location coordinates.

### 2.6 Phylogeography with neutral markers

To establish the phylogenetic relationships among the metapopulations, we utilized SNAPP (Bryant et al., 2012) to create a species tree in BEAST v2.4.0 (Bouckaert et al., 2014) using a coalescent model for unlinked biallelic SNPs. A similar approach to Younger et al. (2017) was used to decrease the computational demand by randomly selecting two individuals per population. Mutation rates were calculated as part of the MCMC using the default parameters. The MCMCs were run for 5 million iterations and convergence was checked in Tracer v1.6 (Drummond & Rambaut, 2007). An effective sample size (ESS) >200 is an acceptable value, and all ESS values were checked to be at least 200. Five thousand and one consensus species trees were generated using TreeAnnotator v2.7.3 (Drummond & Rambaut, 2007) with a 10% burn‐in using and median tree heights. To visualize the resulting species tree, we used Figtree v.1.4.4 (Rambaut, 2018).

### 2.7 Species distribution modeling for mid‐Holocene and LGM

For our analyses of potential glacial refugia for the mountain bluebird, we focused on the Pacific Northwest and Interior Southwest. We defined the Pacific Northwest region to include the intermountain regions between the eastern Cascades and the western Rockies (Figure 1, red) found in Washington, Oregon, and Idaho. We defined the Interior West region to include the various plateaus and basins found throughout the southern Rockies, specifically in Nevada, Wyoming, and Colorado. We then used species distribution models (SDMs) to support our inferences regarding refugia during the last glacial maximum (~21 kyBP) and the mid‐Holocene (~6 kyBP). We obtained 58,287 occurrences of mountain bluebirds (collected during the breeding season in June and July) from the Global Biodiversity Information Facility (GBIF; http://data.gbif.org/). To ensure the accuracy of the georeferencing, we excluded occurrences prior to 1970.

Environmental variables were accessed through the WORLDCLIM dataset (v1.4, http://www.worldclim.org/) using the 2.5 min resolution, which consisted of the 19 variables in the BIOCLIM layers (Hijmans et al., 2005) from 1960 to 1990. We used Pearson's correlation test to look for highly correlated variables (*R* > .80) to avoid biasing the SDM. Nine BIOCLIM layers remained after testing (2, 4, 8, 11, 13, 14, 15, 18, and 19). We combined these layers with an elevation variable from BIOCLIM. To improve model performance, we used additional environmental and topographic variables from the ENVIREM dataset using R (Title & Bemmels, 2018). Environmental data for the mid‐Holocene and the last glacial maximum were also available from the WORLDCLIM dataset. We converted all layers to .asc, resampled, extended, and trimmed for consistency using the R packages raster (Hijmans et al., 2015), rgdal (Bivand et al., 2015), and sf (Pebesma, 2018).

To reduce potential bias, we employed SDMtoolboxPRO v0.9.1 (Brown, 2014) in ArcGIS Pro v3.1.2 (ESRI: Redlands, CA) to rarify the occurrence points (set at 30 km between points). The rarified occurrence points were sampled by buffered local‐adaptive convex hull with a buffer distance of 25 km and an alpha of 5 to generate a bias file. A maximum entropy algorithm in MAXENT v3.4.4 (Phillips et al., 2004, 2006) was used to generate SDMs for the current species distribution of mountain bluebirds, which was then projected to the mid‐Holocene and last glacial maximum. 75% of the rarified points were used for training the model and 25% were withheld for testing model validity. An additional 10,000 background points were selected for comparison against the rarified occurrence points to ascertain climate suitability. We ran 15 cross‐validated replicates with a maximum number of 500 iterations and a convergence threshold of 0.00001. We set the regularization multiplier to 0.1 and used only hinge features for each run. To assess overall model performance, we used the receiver operating characteristic curve (AUC) and the omission curve generated in MAXENT.

### 2.8 Identifying causes of balancing selection

The largest subset of markers (12,258 SNPs) was determined to be under balancing selection. To check for population structure using only the markers under balancing selection, a PCoA was generated in GenAlEx v6.5 (Peakall & Smouse, 2006, 2012). A multi‐loci redundancy analysis (RDA) was performed using the R package vegan/2.6.4 (Oksanen et al., 2022). We obtained three variables from the R package bioclim/0.3.0 (Booth et al., 2014) to model the impact of environment on the dataset. The first was mean temperature during the hottest quarter of the year as this is when birds are on the breeding ground. The second was mean precipitation levels during the warmest quarter of the year, as increased precipitation has been correlated with increased nest failure (McArthur et al., 2017). The third variable was elevation. A RDA for each variable was generated, followed by different combinations of two environmental variables, and finally all three. Since RDAs do not allow for missing data, missing genotypes were filled in using the most common genotype following Forester et al. (2018). Each RDA was plotted using ggplot2 (Wickham, 2016). Each RDA triplot used symmetrical scaling to scale the SNPs and individual scores by the square root of the eigenvalues for easier visualization. Candidate genes for local adaptation were selected based on ordination space having a standard deviation of 3.25 (*p* = .001).

### 2.9 Candidate genes under diversifying selection

While separating the neutral markers from those under selection, Bayescan determined three SNPs to be under diversifying selection (in the top 99 percentile). The position of these SNPs was obtained using Bayescan v2.1 (Foll & Gaggiotti, 2008). We selected positions listed in the annotated Swainson's thrush reference genome to search for genes located in the three corresponding areas.

## 3 RESULTS

### 3.1 Data processing and filtering

We achieved a sequencing output of 1,038,737,832 total reads. Overall, we achieved a mean read depth of 490× and a mean coverage 23% per chromosome following filtering.

### 3.2 Population analyses with neutral markers

When mean observed and expected heterozygosity for the 6998 neutral SNPs were calculated, western Alberta had the lowest at 0.136 and 0.162 while the Cypress Hills had the highest at 0.255 and 0.355, respectively. Overall, the mean observed and expected heterozygosities were similar when compared within each population, with the notable exception of the Cypress Hills which differed by 0.1 (Table 1). Pairwise *F*_ST_ (for neutral loci only) ranged from 0.013 to 0.186 (Table 2). All pairwise comparisons remained significant following the Benjamini–Hochberg correction to reduce the rate of false positives.

**TABLE 1:** Sampling site information with sample sizes (*N*) and observed and expected heterozygosities (*H*_o_ and *H*_e_, respectively).

Population	Abbreviation	*N*	*H*_o_	*H*_e_
Southern British Columbia	SBC	10	0.21117	0.24945
Pacific Northwest	PNW	22	0.19605	0.22592
Nevada	NV	7	0.24929	0.29183
Western Alberta	WAB	17	0.13617	0.16237
Cypress Hills	CYP	5	0.25495	0.35480
Southeast Rocky Mountains	SER	15	0.19571	0.22510

*Note*: Mean expected and observed heterozygosity across all study populations with sample size ≥5 (SK and CCA excluded as *n* = 2). See Figure 1 for locations.

**TABLE 2:** Pairwise *F*_ST_ visualized as a heatmap between all study populations with sample size ≥5 (SK and CCA excluded as *n* = 2).

	SBC	PNW	NV	WAB	CYP	SER
SBC		***	***	***	**	***
PNW	0.186		***	***	**	***
NV	0.038	0.023		***	**	***
WAB	0.037	0.013	0.048		***	***
CYP	0.062	0.029	0.061	0.079		***
SER	0.029	0.015	0.03	0.026	0.045	

*Note*: All values retained significance following Benjamini–Hochberg corrections for multiple comparisons (Benjamini & Hochberg, 1995). Shading for each value corresponds to relative position (higher values are warmer colours, lower values are cooler colours). *p*‐Values <.05*, <.001**, <.0001***.

**FIGURE 2:**
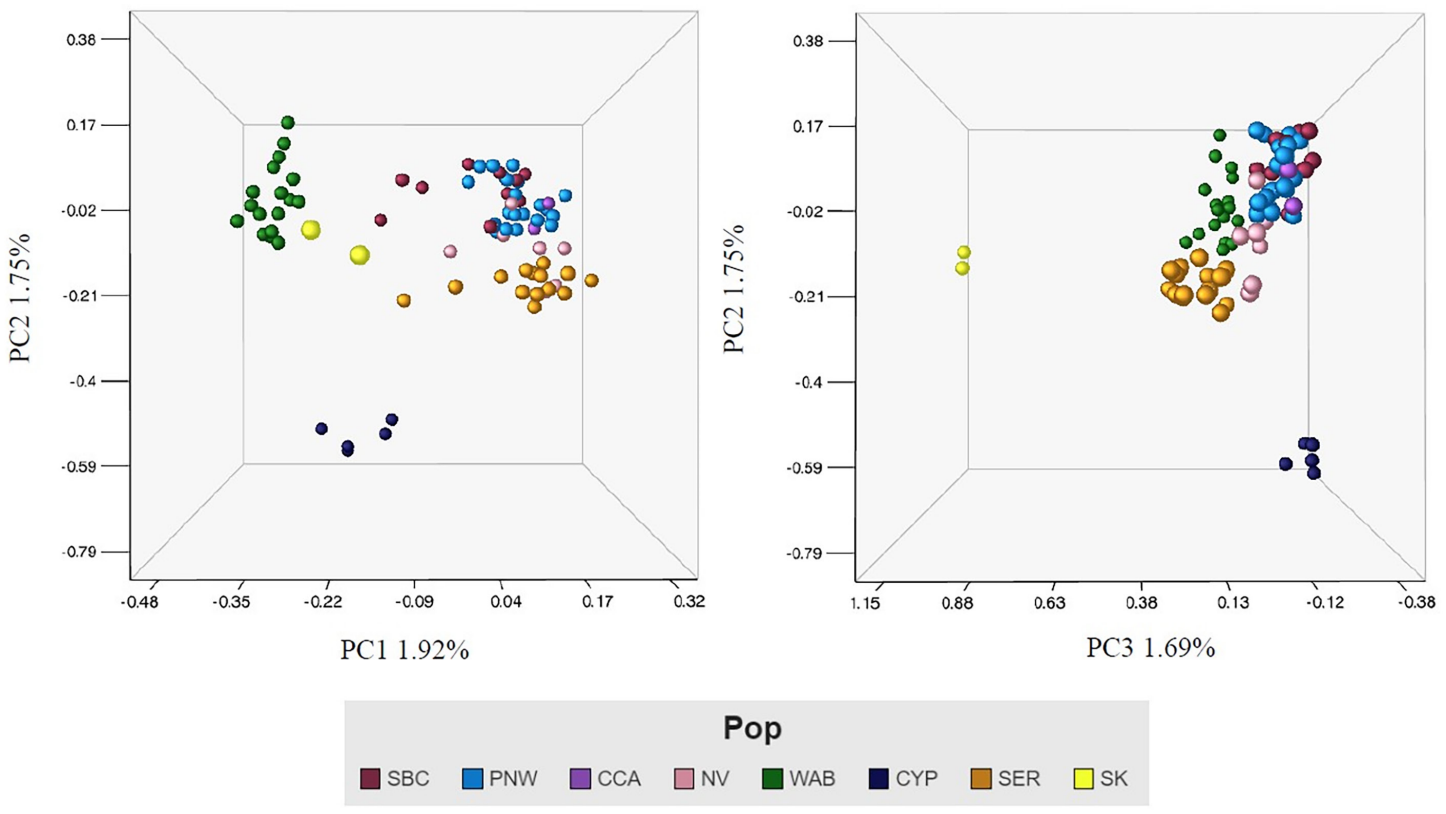
PCoA showing first three PCs accounting for the most variation using neutral markers (*n* = 6998 SNPs) from sites remaining after filtering. Populations correspond as follows: Southern British Columbia (SBC), Pacific Northwest (PNW), Nevada (NV), Western Alberta (WAB), Cypress Hills (CYP), Southeastern Rockies (SER).

The PCoA supported the results of the pairwise *F*_ST_ values, clearly separating birds from western Alberta and the Cypress Hills into their own clusters (Figure 2). The birds from Saskatchewan also formed a distinct cluster, although sample size was small (*n* = 2). The individuals from the four remaining populations, southern British Columbia, the Pacific Northwest, Nevada and the southeastern Rockies, do segregate by locality, but create a larger overlapping cluster. The first three PC accounted for similar amounts of variation with PC1 accounting for 1.92%, followed by the second and third accounting for 1.75% and 1.69%, respectively.

**FIGURE 3:**
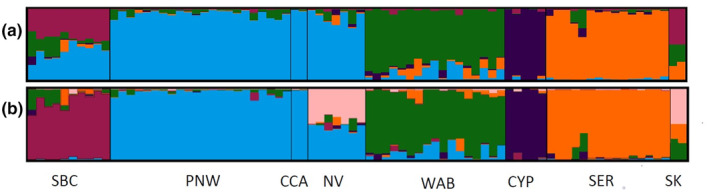
Ancestry matrices based on 80 individuals from eight populations using 6998 SNPs. (a) STRUCTURE Plot (*K* = 5). (b) STRUCTURE plot (*K* = 6). Populations correspond as follows: Southern British Columbia (SBC), Pacific Northwest (PNW), Nevada (NV), Western Alberta (WAB), Cypress Hills (CYP), Southeastern Rockies (SER).

Lastly, the ancestry matrix we generated using STRUCTURE showed the best clustering when *K* = 6 (Figure 3), as supported by the highest ln Pr (*X*|*K*) using the Evanno method (Evanno et al., 2005). Southern British Columbia, western Alberta, the Cypress Hills, and southeastern Rockies each formed their own cluster while the Pacific Northwest and central California formed a single cluster. Nevada and Saskatchewan both showed evidence of admixture. Saskatchewan individuals showed a genetic affinity of 0.5 with a sixth cluster, followed by 0.2–0.25 shared ancestry with southeastern Rockies and 0.25–0.3 with western Alberta. Nevada individuals had about 50% ancestry to a sixth cluster and 50% to the PNW and CCA cluster. Overall, we found a weak but significant relationship (*p* = .010, *R* ^2^ = .0219) between genetic distance and geographic distance (Figure S1). There were no obvious outliers along the slope of the trendline.

### 3.3 Phylogeographic analysis with neutral markers

**FIGURE 4:**
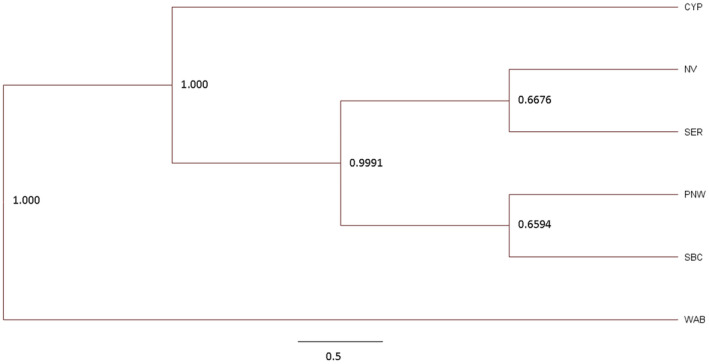
Maximum clade credibility tree using median height from 4500 retained unrooted trees for all study populations with sample size ≥5 (SK and CCA excluded as *n* = 2). Topology based on two random with bootstrap values generated for each node. Populations correspond as follows: Southern British Columbia (SBC), Pacific Northwest (PNW), Nevada (NV), Western Alberta (WAB), Cypress Hills (CYP), Southeastern Rockies (SER).

We visualized the phylogenetic relationships using an unrooted maximum likelihood tree (Figure 4). Both the first and second nodes are strongly supported, with posterior probability of 1. These initial splits separate the western Alberta, followed by the Cypress Hills population, suggesting they are more distantly related to the remaining study populations. An east/west divide becomes apparent in subsequent nodes; the closest sister populations being south British Columbia and the Pacific Northwest (including the two individuals from California) and Nevada with the southeastern Rockies.

### 3.4 Species distribution modeling

Following rarefication, we were left with 3652 occurrence points for the model. All iterations of the model performed better than random (AUC = 0.5) with a mean AUC score of 0.83 ± 0.004. The omission rate closely mirrored the predicted omission as expected. Potential evapotranspiration during the coldest quarter of the year contributed the most to the model, followed by maximum temperature during the coldest month of the year, followed by elevation.

**FIGURE 5:**
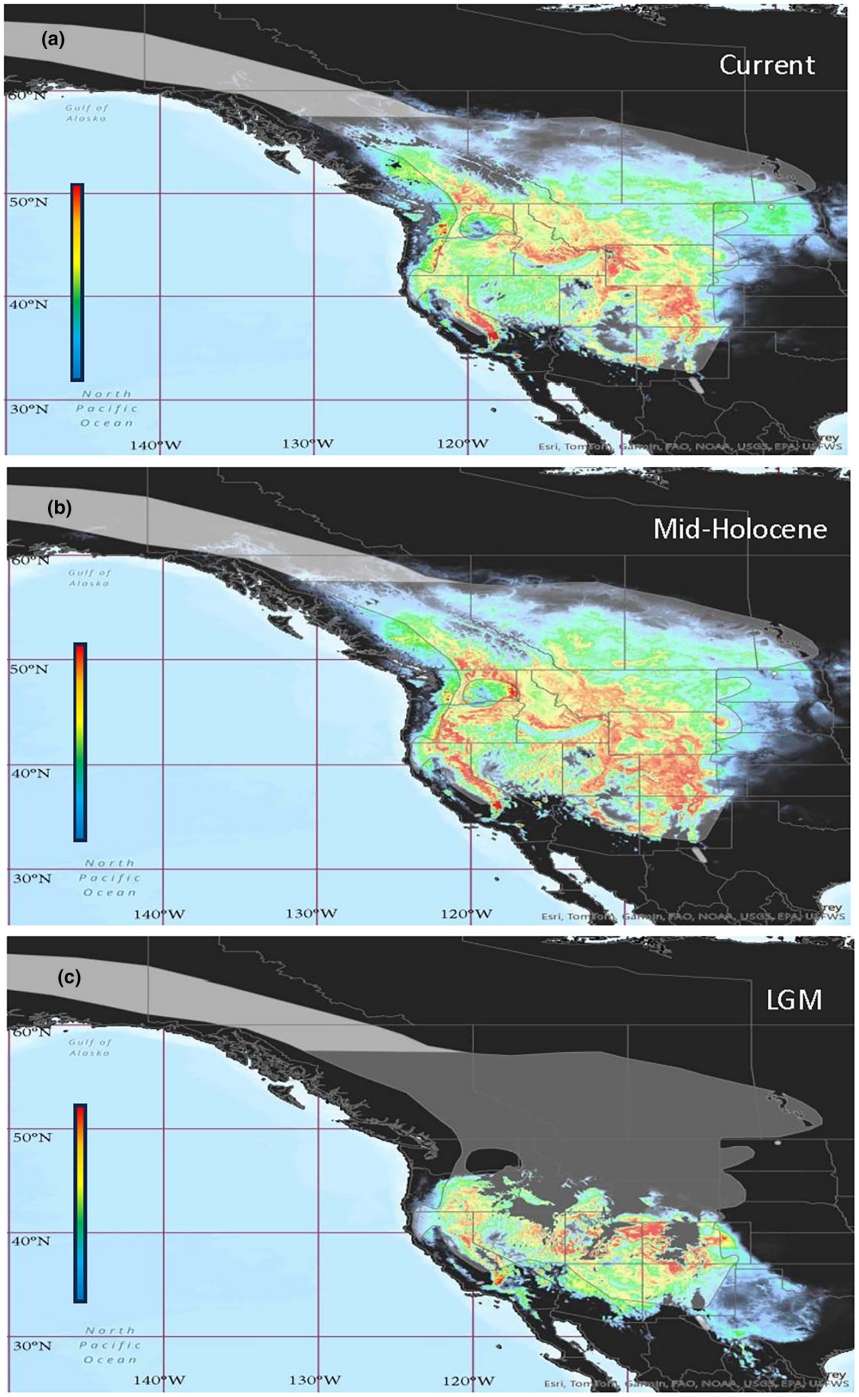
Species distribution models (SDM) created using SDM toolbox (Brown, 2014) in ArcGIS® and MaxEnt (Phillips et al., 2006). Occurrence data for breeding season obtained from Global Biodiversity Information Facility (GBIF; http://data.gbif.org/) along with environmental layers for each time period during the breeding season. Each SDM shows areas where the environmental conditions are/were suitable for the mountain bluebirds to occur. Scales are cumulative and measure percent likelihood of habitat suitability based on the model variables. (a) Current habitat suitability; (b) suitable habitat during the mid‐Holocene; (c) suitable habitat during the LGM.

The species distribution model for current suitable breeding habitat closely resembled the known breeding range of the mountain bluebird (Figure 5a), with some notable suitable habitat in the Appalachian Mountains outside of the known distribution. The mid‐Holocene projected distribution (Figure 5b) also mirrors the current breeding distribution suggesting most breeding populations were already in place by around ~6 kyBP. The LGM projected distribution (Figure 5c) shows a reduced range, mostly concentrated in the southern Pacific Northwest and the interior southwest of the continent.

### 3.5 Redundancy analyses using balancing markers

**FIGURE 6:**
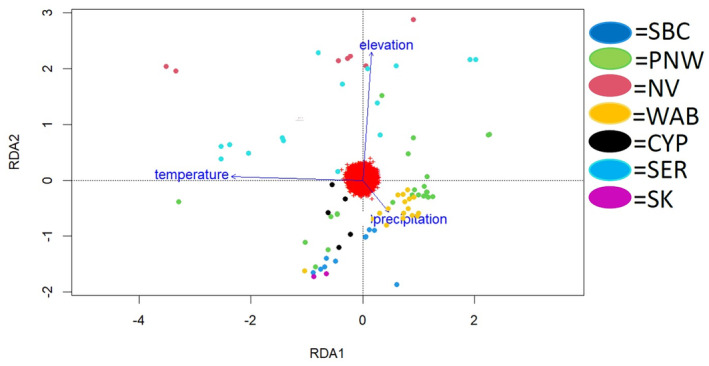
Triplot using all three environmental variables. Although the association between SNPs and the variables were not significant, using three variables in combination divided the northern and southern populations.

All RDA models using the three environmental variables failed to account for the variance within the markers under balancing selection. Seven markers associated with temperature were found to be above the strict 3.25 SD threshold (*p* ≤ .001). When visualized as a triplot, none of the models were able to resolve discrete structure between populations based on the environmental variables used, only the plot with all three variables showed a geographic pattern with a north/south split (Figure 6).

The three markers identified as being under diversifying selection and the seven balancing markers associated with temperature were manually indexed and determined using a subset of Swainson's thrush annotated reference genome originally used for alignment (Table 3).

**TABLE 3:** Three markers and their corresponding candidate genes noted as being under diversifying selection based on global *F*_ST_ scores found in Bayescan.

Chromosome	SNP position	*F*_ST_	Candidate gene	Selection	Function in prior bird studies
1	39,844,386	0.44744	NFKB inhibitor interacting Ras‐like 1	Diversifying	Correlated to immune response during migration
6	468,964	0.29073	Isocitrate dehydrogenase (NAD(+)) 3 catalytic subunit alpha	Diversifying	Unknown
11	6,768,863	0.29037	Aryl hydrocarbon receptor‐like	Diversifying	Unknown
6	21,616,044	0.030335	Neurexin‐3	Balancing	Associated with immune response against avian flu
6	52,958,759	0.030329	SCUBE2	Balancing	Unknown
12	13,513,798	0.030325	SCAPER	Balancing	Unknown
17	12,393,077	0.030321	MYH7B	Balancing	Myocardial formation in embryos
*Z*	14,800,422	0.030329	MRPS30	Balancing	Ribosomal housekeeping gene

### 3.6 Candidate genes under diversifying selection

The first diversifying SNP identified was located at position 39,844,386 on Chromosome 1. When indexed, this SNP was determined to be within the NFKB inhibitor interacting Ras‐like 1 gene. The second SNP was located at position 468,964 Chromosome 6 and corresponds to the gene responsible for the isocitrate dehydrogenase (NAD(+)) 3 catalytic subunit alpha. The final marker was located at position 6,768,863 on Chromosome 11. This position was found to correspond to the aryl hydrocarbon receptor‐like gene.

### 3.7 Candidate genes under balancing selection

Of the seven SNPs potentially responding to temperature, five were linked to known genes (Table 3). Two SNPs were located on Chromosome 6 at positions 21,616,044 and 52,958,759. The first was mapped within the Neurexin‐3 gene and the second was within the SCUBE2 gene. The next marker was on Chromosome 12 at position 13,513,798 and was mapped to the SCAPER gene. Another marker was mapped to Chromosome 17 at position 12,393,077 corresponding to the MYH7B gene. The final marker was located to the Z chromosome at position 14,800,422 to the MRPS30 gene.

## 4 DISCUSSION

Our study provides a novel assessment of the population structure within mountain bluebirds via SNPs examined across the breeding population of these high‐elevation, high‐latitude songbirds. We found various factors, including isolation‐by‐distance, discontinuous habitat, and the northern Rockies, limit connectivity and thus gene flow among contemporary mountain bluebird populations. In regions like Alberta, suitable habitat (Figure 5a, warm colors) is concentrated in the western montane regions of the province and becomes sporadic and disconnected with the start of the dry grassland ecoregion of the Great Plains. Only hotspots of suitable habitat, such as the Cypress Hills in Alberta/Saskatchewan, or the Black Hills of South Dakota, punctuate wide swaths of unsuitable habitat (Figure 5a, cool colors), likely inhibiting gene flow. We also found that the Rocky Mountains do not form a consistent barrier in the American southwest according to our species distribution models and evidence of nesting at high elevation (Haecker, 1948). Furthermore, gene flow on either side of the southern Rockies suggests that suitable valleys and other corridors exist between breeding groups in contrast to what is found in other species, such as greater sage‐grouse (Oyler‐McCance et al., 2005).

When examining historic patterns of gene flow, our LGM models found the majority of suitable mountain bluebird habitat throughout the Pacific Northwest and interior southwest. Although ice‐free corridors likely existed east of the Rockies in Alberta (Beatty & Provan, 2010; Bednarski & Smith, 2007; Dalton et al., 2023; Dyke et al., 2002; Golden & Bain, 2000; Shafer et al., 2010), a putative southwestern Albertan refugia did not appear to have suitable habitat for mountain bluebirds. Likewise, our models also failed to support a refugia located in Beringia. Lastly, we identified five putative candidate genes under balancing selection and three under diversifying selection. Candidate genes under balancing selection were SNPs corresponding to immune response rather than those linked directly to environmental variables.

### 4.1 Population structure and contemporary barriers to gene flow

Mountain bluebirds are widespread throughout discontinuous habitat across western North America (Johnson & Dawson, 2020). As predicted based on their expansive range, we found a significant but weak positive correlation between the geographic distance and genetic distance in mountain bluebird populations. Our species distribution model showed suitable habitat connecting southern British Columbia to both central Washington and Idaho. Isolation‐by‐distance may help to explain why sites with no apparent physical barriers other than distance (e.g., southern British Columbia and the Pacific Northwest) appear distinct in some analyses.

Our second prediction that the Rocky Mountains and discontinuous habitat isolate breeding sites and contribute to population structuring is also supported with the presence of at least four distinct clusters. The Pacific Northwest (Washington, Oregon, Idaho, and central California) is separated from populations east of the Rocky Mountains: western Alberta (southwestern Alberta and central Alberta); the Cypress Hills; and the southeast Rockies (southeast Montana, Wyoming, and Colorado). The Rocky Mountains, especially the northern Rockies, are known to restrict gene flow in other songbirds such as yellow warblers (*Setophaga petechia*) (Milot et al., 2000) and mountain chickadees (*Poecile gambeli*) (Hindley et al., 2018; Spellman et al., 2007; Srikanthan & Burg, 2024), as well as birds considered to be highly mobile like red‐tailed hawks (*Buteo jamaicensis*) (Hull et al., 2009). One possible explanation may be the difference in tree line elevation. The central (e.g., Wyoming) and southern (e.g., Colorado) Rockies have forests at higher elevations (~3000 m and ~ 3500 m, respectively) (Körner, 1998) and thus available nesting habitat at higher elevations. Comparatively, the northern Rockies (British Columbia, Alberta) have a much lower tree line elevation of ~2400 m (Körner, 1998). Populations in the southeastern Rockies may therefore be able to use treed mountain valleys and meadows to disperse around inhospitable mountain peaks while birds in the northern‐most populations (e.g., south British Columbia and western Alberta) cannot. Our SDM for modern day supports this explanation, with most of the suitable habitat for mountain bluebirds concentrated in the southern and central portions of the Rockies.

Discontinuous habit may have the largest effect on the Cypress Hills population, which we also found to be a distinct genetic group despite the proximity of other nearby populations (< 300 km) in western and central Alberta as well as those in Montana, Wyoming, and Colorado. The Cypress Hills are an elevated (~1250 m) region surrounded by semi‐arid grasslands on all sides (Sauchyn, 1990). Previous studies found disjunct populations of western bird lineages, including warbling vireos (*Vireo gilvus*) (Carpenter et al., 2022) and white‐crowned sparrows (*Zonotrichia leucophrys*) (Chilton, 2003) as well as unique genetic lineages of red crossbills (*Loxia curvirostra*) (Benkman, 1999) and ovenbirds (*Seiurus aurocapilla*) (Haché et al., 2017) in the Cypress Hills. Within our modern species distribution model, the Cypress Hills appear as an island of suitable habitat surrounded by a sea of unsuitable habitat. The characteristic xeric grassland of eastern Alberta and southern Saskatchewan that surround the hills offer little in the way of nesting sites and act to separate the remaining suitable habit in western Alberta, acting as another barrier to gene flow.

### 4.2 Glacial refugia, expansion, and recolonization post‐LGM

Like many species, mountain bluebirds were displaced from their northern range by the ice sheets during the last glacial maximum. Our species distribution model for this period found no suitable habitat north of the 49th parallel, ruling out far northern refugia like Beringia. The lack of suitable habitat is not surprising, however, seeing as the ice sheets stretched into northern Washington, Idaho, and Montana in the west (Shafer et al., 2010) covering most of mountain bluebirds' northern range.

**FIGURE 7:**
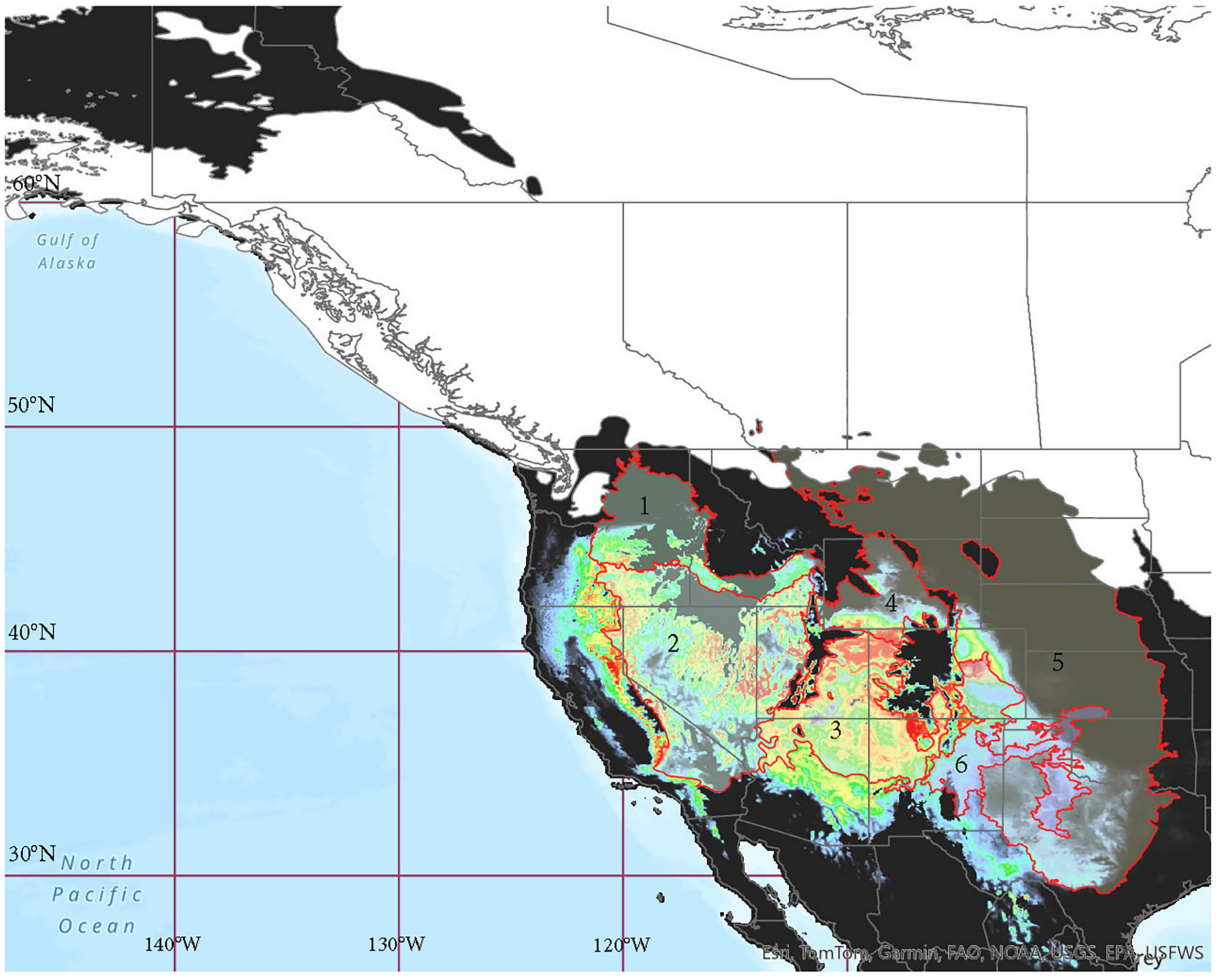
Proposed glacial refugia for mountain bluebirds during the LGM based on the corresponding species distribution model. Extent of LGM ice sheets obtained from Dalton et al. (2022) shown in white. Current geographic regions outlined in red: 1. Columbia Basin, 2. Great Basin, 3. Colorado Plateau 4. Wyoming Basin, 5. Great Plains, 6. Southwest Tablelands.

We found that the Pacific Northwest, Nevada, and the southeastern Rockies are likely the oldest populations of mountain bluebirds of which we sampled, based on the existence of suitable habitat as shown by our SDM during the LGM (Figure 7). Given their affinity for nesting in stands of trembling aspen (Johnson & Dawson, 2020), it appears that refugia suited for aspen may have doubled as refugia for mountain bluebirds. During the last glacial maximum, trembling aspen had three main populations: one in the coastal Cascades; a second spanning the eastern slope of the Cascades, through to the Sierra Nevadas and the northern Rocky Mountains; and a third along the eastern US Rocky Mountains (Montana, Wyoming, and Colorado) and eastern United States (Bagley et al., 2020). The second and third populations of trembling aspen fit well with the structuring seen in mountain bluebirds and would have allowed for the connectivity observed over long distances. Our species distribution model also supports aspen refugia corresponding to our structure with suitable environmental conditions concentrated in the intermountain regions within the Columbia Basin and Great Basin and the southwest Tablelands and High Plains.

Alternatively, the population structure and increased genetic diversity observed in certain populations (e.g., the Pacific Northwest) may be explained by hybridization (Grant & Grant, 2019). Southern British Columbia, the Pacific Northwest, and the southern portion of the southeastern Rockies (specifically Colorado) all fall within the current breeding range of the western bluebird. If a temporary and dynamic hybrid zone resulted from range expansion between the two species, as one currently has in western Montana (Duckworth & Semenov, 2017), it likely would have been at the end of the LGM as populations began to expand from their refugia.

Despite being west of the Rocky Mountains, Nevada was intermediate in the PCoA between the Pacific Northwest and the southeastern Rockies. Furthermore, Nevada showed admixture with the Pacific Northwest in both ancestry matrixes. The phylogeographic analysis, however, placed Nevada as the sister group to the southeastern Rockies. Our species distribution model shows Nevada would have contained a mosaic of suitable habitat for mountain bluebirds during the last glacial maximum, mostly concentrated in the southwest and the northeast portion of the state. This split supports mitochondrial evidence in other species where western individuals were genetically similar to those in the Columbia Basin while eastern individuals within the Colorado Plateau grouped with the Wyoming Basin and the Great Plains (Figure 7, Riddle & Honeycutt, 1990). Nevada may therefore represent an area of secondary contact between the Pacific Northwest and the southeastern Rockies.

During the last glacial maximum, Alberta was covered by the Laurentide ice sheet, as was most of Canada and the northern United States east of the Rockies (see full extent in Dalton et al., 2022). If a putative glacial refugium did exist in southwestern Alberta, our species distribution model did not show this as suitable habitat at the time. However, pollen profiles show that as early as 14 kyBP, the Cypress Hills and portions of southern Alberta were possibly connected to each other by Cordilleran forest with either shrubland or a mixture of *Populus/Salix/Poaceae* parkland to the south (Strong & Hills, 2005). Both western Alberta and the Cypress Hills were likely among some of the first locations to be repopulated by expanding bluebird populations. However, where these founding populations came from is less clear. Despite being east of the Rockies, neither the western Alberta population nor the Cypress Hills population appear to cluster closely to any other population in our PCoA (Figure 2). Interestingly, our ancestry matrix (Figure 3) does show shared ancestry with the Pacific Northwest, southern British Columbia, and the southeastern Rockies for a number of individuals within western Alberta. The ancestry matrix for western Alberta also shows shared ancestry with the Cypress Hills, yet the Cypress Hills appear to show very little ancestry with western Alberta. By 12 kyBP, the area around the Cypress Hills transitioned to less suitable xeric grasslands, ultimately isolating the sky island from the rest of the Cordilleran forest in the west and south (Strong & Hills, 2005).

### 4.3 Genes of interest under balancing and directional selection

We detected around 12,200 SNPs under balancing selection—a signal associated with high‐allelic diversity (Fijarczyk & Babik, 2015). High‐allelic diversity may be maintained by factors including habitat heterogeneity leading to local adaptation, overdominance or heterozygote advantage, and frequency‐dependent selection (Fijarczyk & Babik, 2015). Studies involving avian candidate genes have typically been focused on genes associated with migration or plumage morphology (e.g., Chakarov et al., 2013; Peterson et al., 2013; Ramos et al., 2017; Walsh et al., 2012). Of the candidate genes, we identified based on the associated SNPs (Table 3), only a few have received attention in bird‐based study systems (primarily in domestic chickens, *Gallus gallus* var. *domesticus*). The Neurexin‐3 gene, for example, was associated with survivorship following outbreaks of the HPAI H7N2 virus in commercial laying chickens (Drobik‐Czwarno et al., 2018). As viruses may vary based both on environmental conditions and the biota present (Morin et al., 2018), the diversity in this gene may be maintained by environmental conditions and specific species the different mountain bluebird populations interact with.

With mountain bluebirds experiencing a large range of temperatures during the breeding season, those in warmer environments are likely at greater risk of heat stress than individuals elsewhere in the range. Therefore, selection would likely favor individuals capable of mitigating heat stress. The MYH7B gene is involved in the development of embryonic chicken hearts, specifically, the development of the myocardium (Warkman et al., 2012). In rock pigeons (*Columba livia*), heat acclimation induces alterations in myocardial beta‐adrenoreceptor (Arieli & Marder, 1998). Although not studied in bluebirds, perhaps a similar mechanism occurs whereby populations in warmer climates have acclimatized to heat stress, causing similar alterations to the development of their myocardium. Likewise, the MRPS30 gene is a ubiquitous housekeeping gene with the highest activity in the heart and muscles and codes for ribosomal subunit proteins (Davies et al., 2012). The expression levels of MRPS30 also remain relatively stable in birds undergoing heat stress (Cedraz de Oliveira et al., 2017; Gromboni et al., 2020). Maintaining the proteins responsible for assisting with the folding of other proteins would be critical at high temperatures, thus variation within the overall population may be maintained by differences in breeding site temperature.

The three candidate genes under diversifying selection have also received little attention in avian model systems, with only one gene being directly studied in songbirds. The NFKB inhibitor interacting Ras‐like 1 gene is an orthologous gene not yet understood within humans (Murphy et al., 2006). It belongs to a family of immune system genes that are functionally inactive through inhibition until the phosphorylation of the inhibitor gene and subsequent cleavage (Yamamoto & Gaynor, 2004). Previous work in the red‐headed bunting (*Emberiza bruniceps*) has suggested that NFKB genes play an important role in defense against the various pathogens encountered and can be transmitted during migration (Tiwari et al., 2023). Populations of mountain bluebirds undergoing more strenuous migrations (such as the populations in northern Canada) may also experience differences in the pathogens they encounter; therefore, this would place the corresponding alleles of this gene under directional selection.

One of the other genes under diversifying selection in mountain bluebirds, NAD(+), also has an ortholog in humans. NAD(+) encodes the alpha subunit protein of an isozyme of NAD(+)‐dependent isocitrate dehydrogenase (Murphy et al., 2006), although again its role in birds remains unknown. The last gene, aryl hydrocarbon‐like receptor gene, is responsible for gene regulation of metabolic enzymes, immune response, stem cell maintenance, and cellular differentiation in humans (Gutiérrez‐Vázquez & Quintana, 2018).

## 5 CONCLUSIONS

Our study provides a population‐wide examination of mountain bluebird population genetic structure across their range. Using neutral markers, we found consistent support for at least four breeding groups (consistently of the Pacific Northwest, western Alberta, Cypress Hills, and the southeastern Rockies). The smallest of these groups was found in the Cypress Hills, where both relict and unique bird lineages have been found in the past. Both our species distribution models, and genetic analyses support the northern Rocky Mountains as a barrier to connectivity, but further south in more temperate climates such as the southeastern Rockies, they allow for gene flow. This was also the case during the last glacial maximum, when mountain bluebirds would have been concentrated in areas of suitable habitat on either side of the Rocky Mountains within the Pacific Northwest (namely the intermountain regions) and the southern Rockies where open aspen parkland and subalpine forests were predominant. To get a better understanding of population structure within mountain bluebirds and their genetics, samples from the extreme north and extreme south, as well as additional samples from peripheral populations in Saskatchewan, are likely required as well as the addition of whole‐genome sequencing techniques. Lastly, we identified three candidate genes under balancing selection and five under diversifying selection. In terms of balancing selection, we found SNPs corresponding to immune response rather than those linked directly to environmental variables. Overall, our study has provided valuable insights into the connectivity and genetic variation present in mountain bluebirds across most of their range. The complex population structure seen in mountain bluebirds outlines possible barriers for other migratory species found at high elevations and high latitudes. Our study also emphasizes the importance of habitat connectivity and suitability, even for species that have been able to use and capitalize on human‐altered habitat.

## AUTHOR CONTRIBUTIONS

**Aaron Veale:** Conceptualization (equal); data curation (lead); formal analysis (lead); investigation (supporting); methodology (equal); writing – original draft (lead). **Matthew W. Reudink:** Conceptualization (equal); funding acquisition (equal); project administration (equal); supervision (equal); writing – review and editing (equal). **Theresa M. Burg:** Conceptualization (equal); funding acquisition (equal); methodology (equal); project administration (equal); resources (lead); supervision (equal); writing – review and editing (equal).

## CONFLICT OF INTEREST STATEMENT

The authors declare no competing interests.

## Supporting information


Table S1.

## Data Availability

The sequences, VCF files, and supporting files can be found at the following: https://doi.org/10.5061/dryad.rxwdbrvgm.
