# Supplementary material for: Neutral markers reveal complex population structure across the range of a widespread songbird
Source: Ecol Evol. 2024 Jul 7;14(7):e11638. doi: 10.1002/ece3.11638 (PMC11228359; doi:10.1002/ece3.11638)
Supplement: Supplementary file 1 — Table S1. [file ECE3-14-e11638-s001.zip › sm_0001-Supinfo.docx]

**Supplemental Table 1 –** Collection information for samples used in analyses, organized by population. *As Central California was found to group genetically with the Pacific Northwest in both the PCoA and the ancestry matrix, the two individuals were grouped with the Pacific Northwest for the remaining analyses.

**Supplemental Figure 1 -** Mantel test for genetic distance as a response variable to geographic distance using neutral marker dataset.
