# Supplementary figures and images for: Neutral markers reveal complex population structure across the range of a widespread songbird
Source: Ecol Evol. 2024 Jul 7;14(7):e11638. doi: 10.1002/ece3.11638 (PMC11228359; doi:10.1002/ece3.11638)

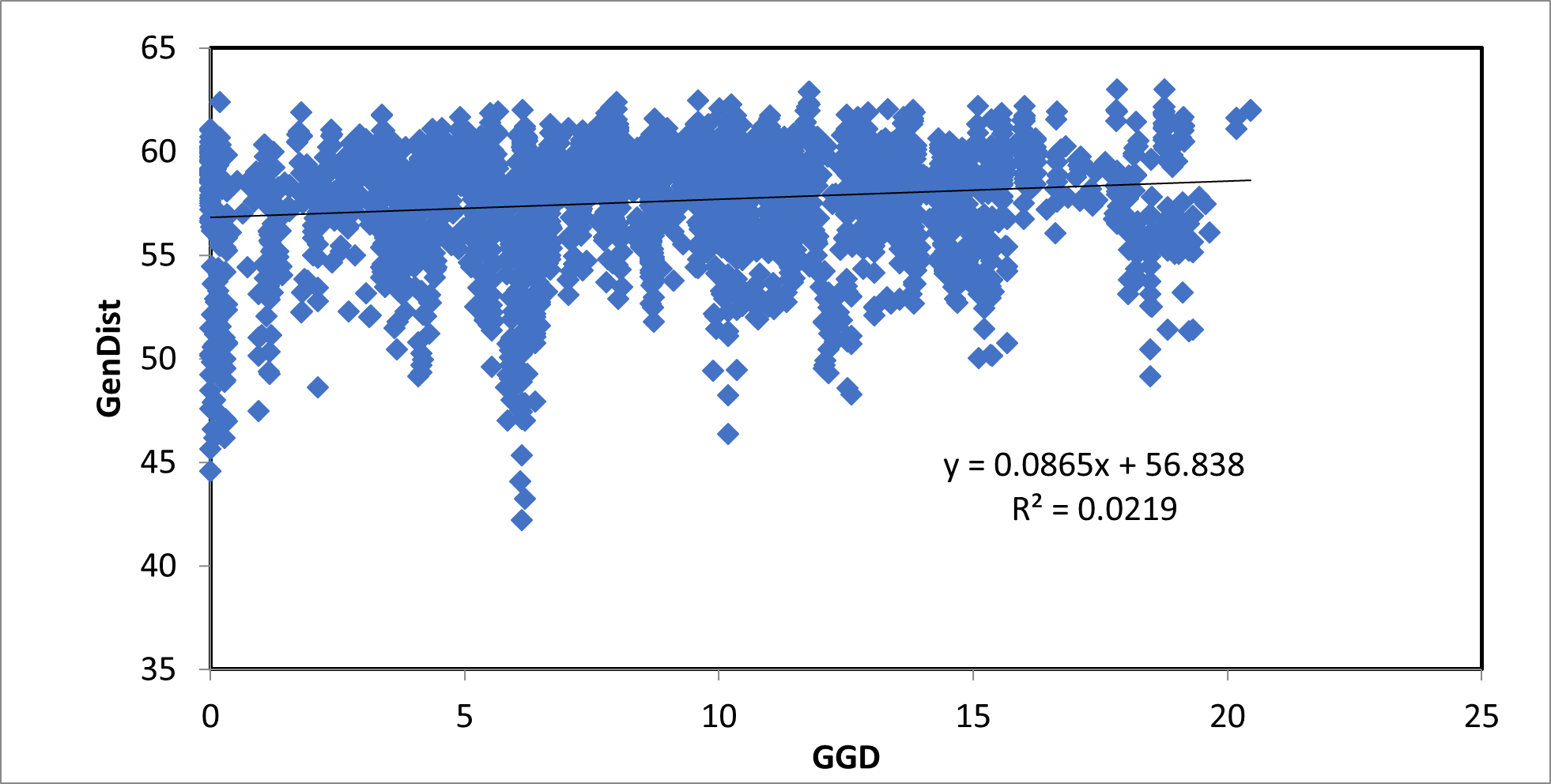

Supplement: Supplementary file 1 — Table S1. [file ECE3-14-e11638-s001.zip › SupplementalFigure1.jpg.png]

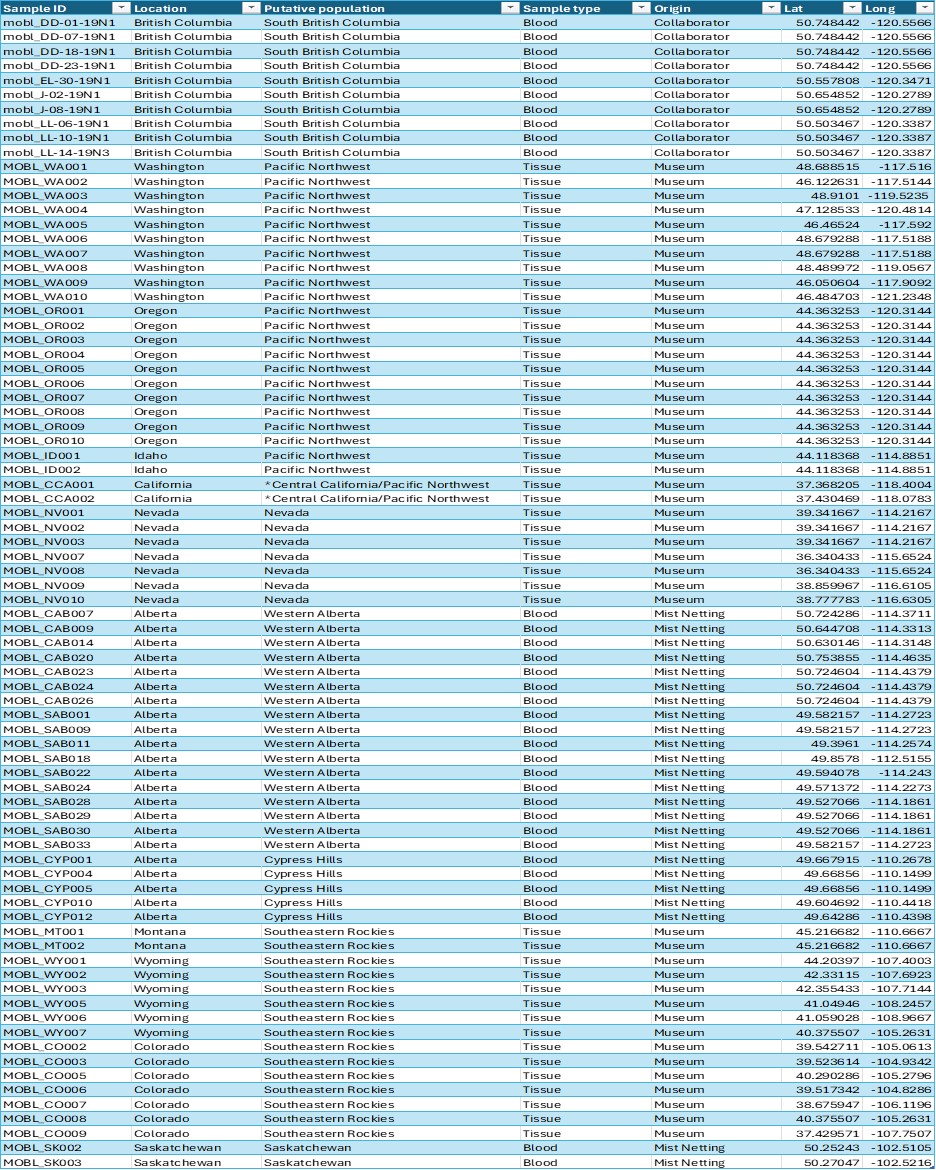

Supplement: Supplementary file 1 — Table S1. [file ECE3-14-e11638-s001.zip › SupplementalTable1.jpg]
